# Analysis of gene expression and chemoresistance of CD133^+ ^cancer stem cells in glioblastoma

**DOI:** 10.1186/1476-4598-5-67

**Published:** 2006-12-02

**Authors:** Gentao Liu, Xiangpeng Yuan, Zhaohui Zeng, Patrizia Tunici, Hiushan Ng, Iman R Abdulkadir, Lizhi Lu, Dwain Irvin, Keith L Black, John S Yu

**Affiliations:** 1 Maxine Dunitz Neurosurgical Institute, Cedars-Sinai Medical Center, Los Angeles, CA, 90048, USA; 2 Division of Hematology/Oncology, Cedars-Sinai Medical Center/David Geffen School of Medicine at UCLA, Los Angeles, CA, 90048, USA; 3 Institute of Animal Husbandry and Veterinary Science, Zhejiang Academy of Agricultural Sciences, Hangzhou, 310021, P.R. China; 4 8631 West Third Street, Suite 800E, Los Angeles, CA, 90048, USA

## Abstract

**Background:**

Recently, a small population of cancer stem cells in adult and pediatric brain tumors has been identified. Some evidence has suggested that CD133 is a marker for a subset of leukemia and glioblastoma cancer stem cells. Especially, CD133 positive cells isolated from human glioblastoma may initiate tumors and represent novel targets for therapeutics. The gene expression and the drug resistance property of CD133 positive cancer stem cells, however, are still unknown.

**Results:**

In this study, by FACS analysis we determined the percentage of CD133 positive cells in three primary cultured cell lines established from glioblastoma patients 10.2%, 69.7% and 27.5%, respectively. We also determined the average mRNA levels of markers associated with neural precursors. For example, CD90, CD44, CXCR4, Nestin, Msi1 and MELK mRNA on CD133 positive cells increased to 15.6, 5.7, 337.8, 21.4, 84 and 1351 times, respectively, compared to autologous CD133 negative cells derived from cell line No. 66. Additionally, CD133 positive cells express higher levels of BCRP1 and MGMT mRNA, as well as higher mRNA levels of genes that inhibit apoptosis. Furthermore, CD133 positive cells were significantly resistant to chemotherapeutic agents including temozolomide, carboplatin, paclitaxel (Taxol) and etoposide (VP16) compared to autologous CD133 negative cells. Finally, CD133 expression was significantly higher in recurrent GBM tissue obtained from five patients as compared to their respective newly diagnosed tumors.

**Conclusion:**

Our study for the first time provided evidence that CD133 positive cancer stem cells display strong capability on tumor's resistance to chemotherapy. This resistance is probably contributed by the CD133 positive cell with higher expression of on BCRP1 and MGMT, as well as the anti-apoptosis protein and inhibitors of apoptosis protein families. Future treatment should target this small population of CD133 positive cancer stem cells in tumors to improve the survival of brain tumor patients.

## Background

Recently, we and other groups have identified a small population of cancer stem cells in adult and pediatric brain tumors [1-4]. These cancer stem cells form neurospheres and possess the capacity for self-renewal. They also express genes associated with neural stem cells (NSCs) and differentiate into phenotypically diverse populations including neuronal, astrocytic and oligodendroglial cells. The novel cell-membrane protein CD133, has been identified as a marker of a subset of neural stem cells in the adult central nervous system as well as of glioblastoma stem-like cells [1,3]. CD133 positive cancer stem cells have a capacity for unlimited self-renewal, as well as the ability to initiate and drive tumor progression in an animal model [1].

We hypothesized that CD133 positive cancer stem cells are likely to share many of the properties of normal stem cells that provide for a long lifespan, including: relative quiescence; resistance to drugs and toxins through the expression of several ABC transporters; an active DNA-repair capacity; and resistance to apoptosis [5]. Bearing properties of normal neural stem cells, we inferred that these cancer stem-like cells may not only give us insight into oncogenesis of glioblastoma but also explain clinical resistance of these tumors to conventional chemotherapeutic agents. Clinically it is observed that tumors respond to chemotherapies only to recur with renewed resilience and aggression. Although chemotherapy kills most of the cells in a tumor, cancer stem cells may be left behind, which then recur be an important due to their chemoresistance. In this study, for the first time we provided evidence that CD133 positive cancer stem cells display significant resistance to conventional chemotherapeutic agents. These features may be correlated to the overexpression of drug resistance genes such as BCRP1 and DNA-mismatch repair genes such as MGMT, as well as genes related to inhibiting cell apoptosis on CD133 positive cancer stem cells. Furthermore, we show that CD133 gene expression is significantly higher in the recurrent GBM tumor tissue from five patients as compared to their respective newly diagnosed tumors. These data suggest that CD133 positive cancer stem cells are resistant to current chemotherapy and may represent a cell target for novel glioblastoma therapies.

## Results

### Isolation of CD133 positive cancer stem cells

**Figure 1:**
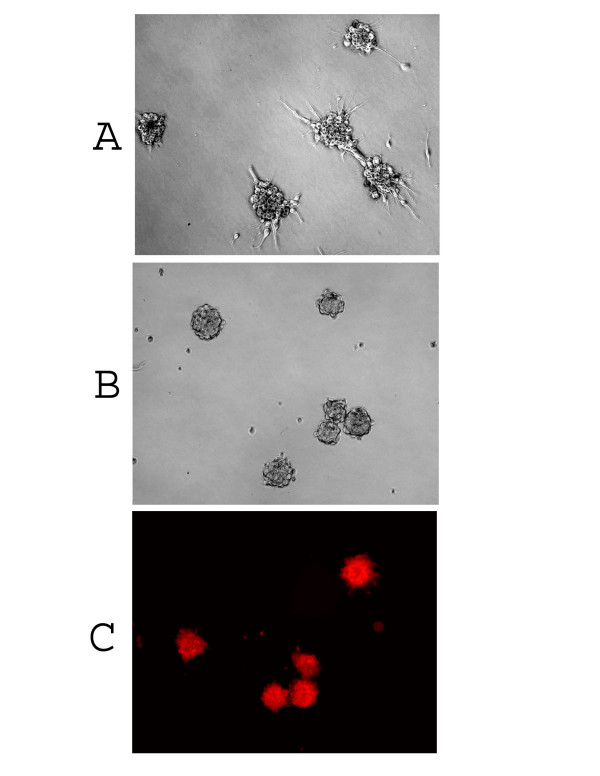
**Primary culture of adult glioblastoma cells**. (A) Tumor cells looking like neurosphere were found in three glioblastoma primary cultured cell lines in 10% FBS/DMEM/F-12 medium. (B) Neurosphere derived from a single isolated CD133 positive cell cultured in NSC medium. (C) CD133 expression on neurospheres derived from a single isolated CD133 positive cell in NSC medium. Red staining indicates CD133 positive. (Magnification = ×100).

**Figure 2:**
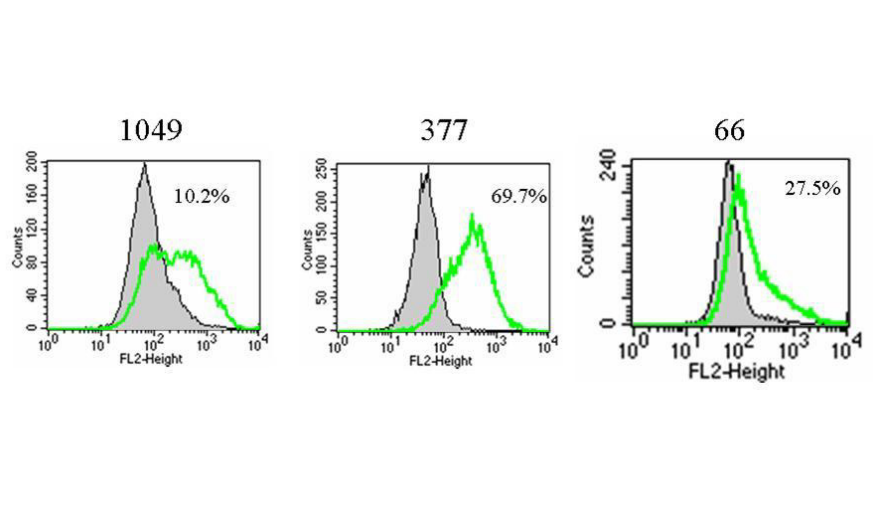
**CD133 protein expression on primary cultured cells**. Tumor cells were cultured in 10% FBS/DMEM/F-12 medium for 3–6 passages and stained with specific mAb to CD133, and isotype control-matched mAb. Results are given as the percentage of CD133 positive cells in the total population. In the histograms, the thick green line represents staining with CD133 mAb, and the gray lines represent the isotype control-matched mAb.

Recently, CD133 has been identified as a marker of the subset of glioblastoma stem cells [1,3]. In this study, after screening thirty glioblastoma patients' primary cultured cells, we found that three glioblastoma patients' tumor cells (No. 66, No. 377 and No. 1049) could form separate colonies in 10% FBS/DMEM/F-12 culture medium for 3–6 passages (Fig 1A), which in turn became floating neurospheres when switching to serum-free medium containing EGF/FGF (NSC medium). Based on our previous report on the characterization of cancer stem cells [2], we assumed some cancer stem cells might have existed in these three primary cultured cells. Because CD133 has been identified as a powerful cancer stem cell marker, we then examined and found CD133 expression that on these three primary cultured cell lines in 10% FBS/DMEM/F-12 medium represented 10.2%, 69.7% and 27.5% of the total population examined on No. 1049, No. 377 and No. 66, respectively, by flow cytometry analysis (Fig. 2). We utilized FACS sorting to isolate CD133 positive and CD133 negative cells from the above three primary cultured cell lines to analyze gene expression and chemoresistance of these two populations. In addition, single isolated CD133 positive cancer stem cell was demonstrated to have the capacity for self-renewal and clonogenic potential (Fig. 1B) and sustain expression of CD133 in NSC medium (Fig. 1C).

### Expression of markers associated with neural precursors on CD133 positive tumor cells

It has been demonstrated that tumors contain CD133 positive cells and that such cells drive tumor progression [1]. The expression of other genes related to cell "stemness" on CD133 positive cells, however, is still unknown. Therefore, we collected the CD133 positive cells and CD133 negative cells by FACS sorting from the three primary cultured cells (No. 66, No. 377 and No. 1049) and used real-time PCR to analyze some markers associated with neural precursors in these two populations. The results were shown as in Table 1 section A. For example, we found that CD90, CD44, CXCR4, Nestin, Musashi-1 (Msi1) and maternal embryonic leucine zipper kinase (MELK) mRNA expression on CD133 positive cancer stem cells derived from No. 66 increased an average of 15.6, 5.7, 337.8, 21.4, 84, 1351 times, respectively, compared to the levels on autologous CD133 negative tumor cells. In addition, we found that mRNA levels for GLI1 and PTCH increased an average of 46 and 16 times, respectively, in CD133 positive cells compared to autologous CD133 negative cells derived from No.66. Similar results were also found in both No. 377 and No. 1049 cell lines (Table 1, section A). Furthermore, we found Bmi-1, phosphoserine phosphatase (PSP), SHH, OCT4 and Snail mRNA expressed in CD133 positive cells derived from above three cell lines, however, none of the five genes were detectable on CD133 negative cells (data not shown).

**Table 1:** Different gene mRNA expression on CD133+ cells compared to those on autologous CD133- cells *

		No. 66		No. 377		No. 1049	
Section	Gene name	CD133-	CD133+	CD133-	CD133+	CD133-	CD133+
A	CD90	1	15.6 ± 0.66	1	12.8 ± 0.94	1	13.5 ± 0.75
	CD44	1	5.7 ± 0.48	1	2.5 ± 0.22	1	2.8 ± 0.19
	CXCR4	1	337.8 ± 29.2	1	251.5 ± 22.1	1	264.9 ± 22.9
	Nestin	1	21.4 ± 1.25	1	23.2 ± 1.65	1	22.1 ± 1.54
	MSI	1	84 ± 7.6	1	75.4 ± 7.03	1	53.5 ± 6.2
	MELK	1	1351 ± 95.8	1	467.7 ± 40.5	1	514.6 ± 45.6
	GLI-1	1	46 ± 3.8	1	43 ± 4.5	1	49 ± 5.9
	PTCH	1	16 ± 1.48	1	13.5 ± 0.85	1	14.3 ± 1.24

B	MGMT	1	32.4 ± 2.5	1	34.7 ± 2.9	1	56.3 ± 4.2
	BCRP1	1	6.5 ± 0.43	1	4.3 ± 0.25	1	4.8 ± 0.24
	SIRT1	1	4.9 ± 0.34	1	4.2 ± 0.26	1	5.4 ± 0.29
	FLIP	1	294 ± 25.5	1	157.6 ± 14.2	1	145.6 ± 13.7
	BCL-2	1	13.9 ± 0.95	1	4.9 ± 0.54	1	3.8 ± 0.54
	BCL-XL	1	5.6 ± 0.39	1	3.2 ± 0.16	1	2.5 ± 0.14
	cIAP1	1	39.0 ± 3.5	1	4.3 ± 0.53	1	5.6 ± 0.65
	cIAP2	1	3 ± 0.25	1	1.9 ± 0.12	1	1.7 ± 0.14
	XIAP	1	21.9 ± 2.2	1	9.7 ± 0.68	1	10.3 ± 0.91
	NAIP	1	12.1 ± 0.75	1	6.4 ± 0.43	1	4.5 ± 0.62
	Survivin	1	1.6 ± 0.08	1	2.3 ± 0.18	1	2.4 ± 0.18
	BAX	1	0.33 ± 0.03	1	0.49 ± 0.06	1	0.21 ± 0.05

* Total RNA was extracted from both CD133+ and CD133- fraction after FACS sorting from three primary cultured cells (No. 66, 377 and 1049). Different gene mRNA expression was measured by real-time qPCR. The relative mRNA expression of different gene in CD133+ cells was presented as the fold increase compared to that of autologous CD133- cells (see Methods). Data was presented as Mean ± SD of three independent experiments.

### Drug sensitivity of CD133 positive cancer stem cells

**Figure 3:**
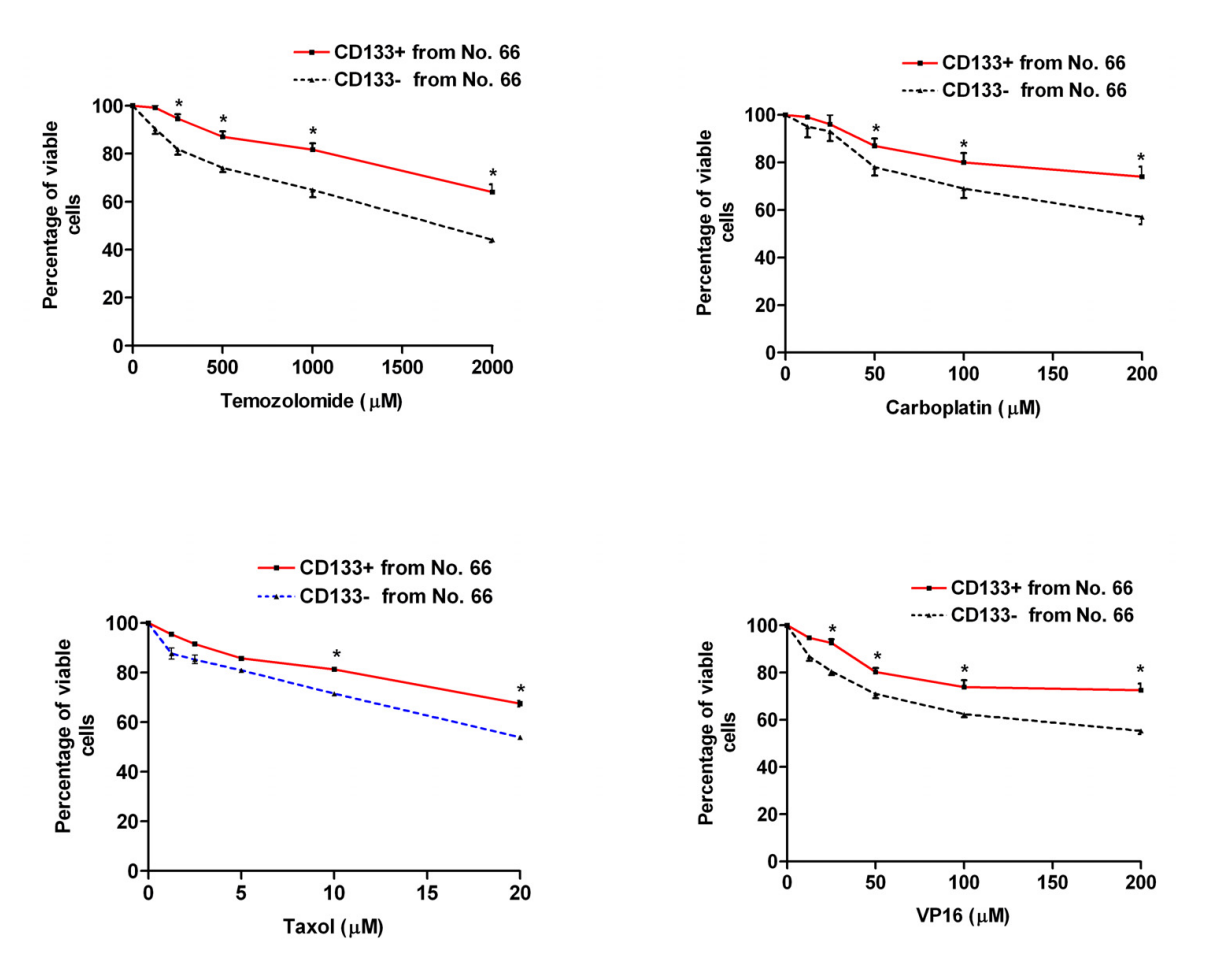
**Drug sensitivity of CD133 positive cancer stem cells derived from No. 66**. Both CD133 positive (CD133+) and CD133 negative (CD133-) tumor cells were collected from No. 66 by FACS sorting. 1 × 10^4 ^cells/well were plated in 96-well plate and treated with various concentrations of temozolomide, carboplatin, Taxol and VP16 for 48 hours in FBS/DMEM/F-12 medium. * indicates p < 0.05 compared to autologous CD133- cells. Data are representative of two independent experiments.

**Figure 4:**
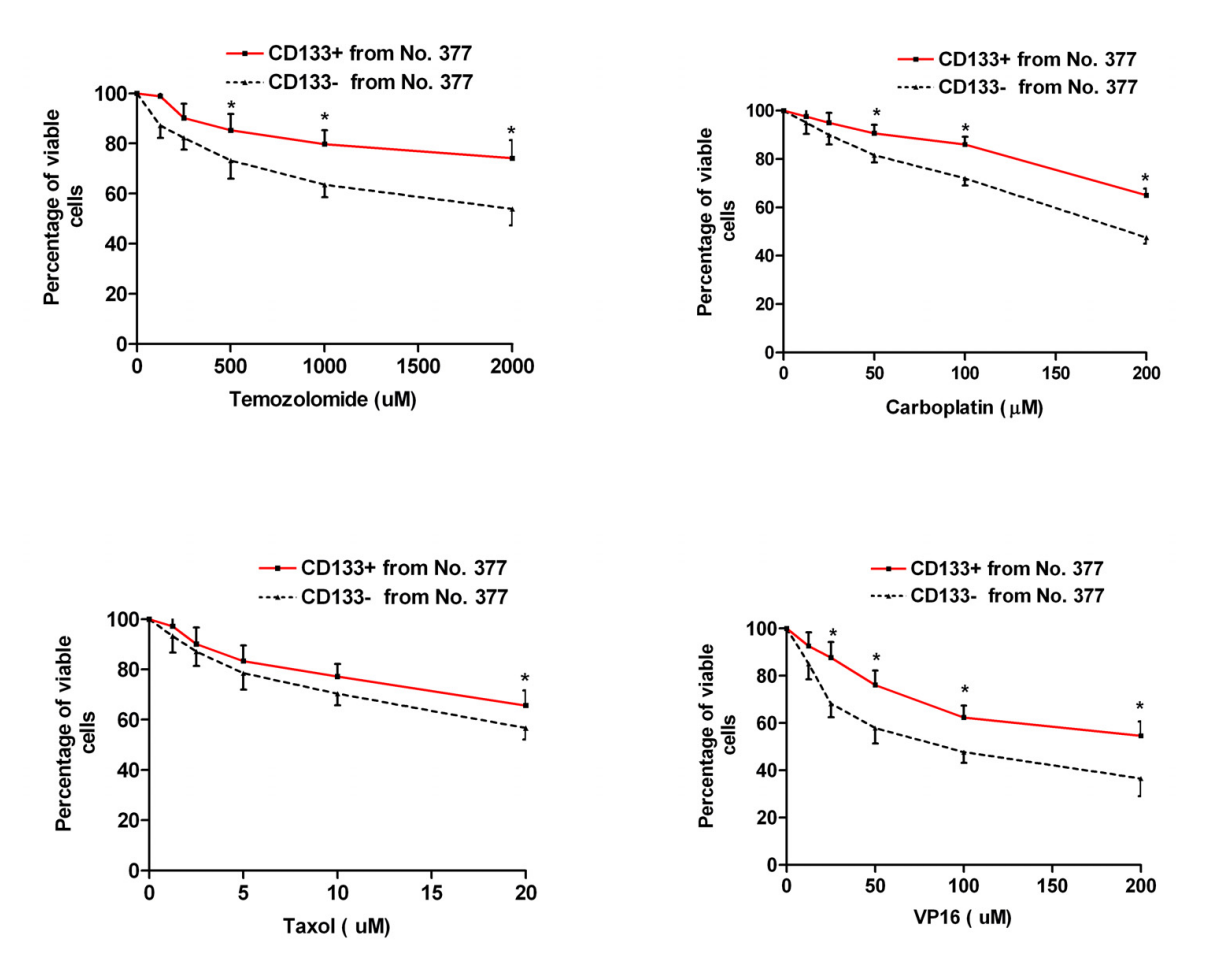
**Drug sensitivity of CD133 positive cancer stem cells derived from No. 377**. Both CD133+ and CD133- tumor cells were collected from No. 377 by FACS sorting. 1 × 10^4 ^cells/well were plated in 96-well plate and treated with various concentrations of temozolomide, carboplatin, Taxol and VP16 for 48 hours in 10% FBS/DMEM/F-12 medium. * indicates p < 0.05 compared to autologous CD133- cells. Data are representative of two independent experiments.

**Figure 5:**
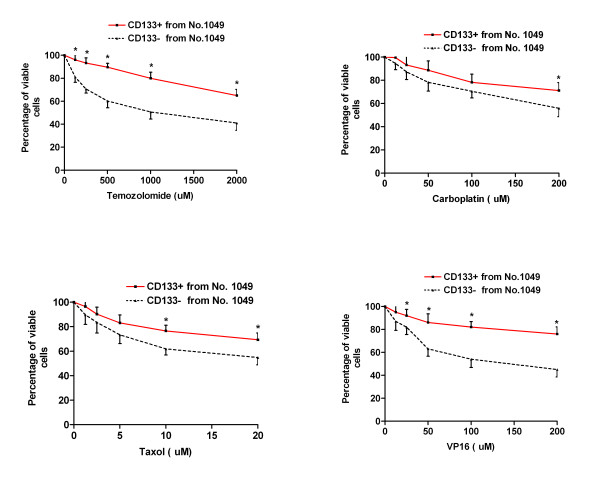
**Drug sensitivity of CD133 positive cancer stem cells derived from No. 1049**. Both CD133+ and CD133- tumor cells were collected from No. 1049 by FACS sorting. 1 × 10^4 ^cells/well were plated in 96-well plate and treated with various concentrations of temozolomide, carboplatin, Taxol and VP16 for 48 hours in 10% FBS/DMEM/F-12 medium. * indicates p < 0.05 compared to autologous CD133- cells. Data are representative of two independent experiments.

At a fundamental level, cancer is resilient to treatment because malignant cells survive chemotherapy and radiation, or avoid the immune surveillance of endogenous cytotoxic T cells and natural killer cells. Cancer stem cells have a capacity for unlimited self-renewal, as well as the ability to initiate and drive tumor progression in an animal model [1]. We hypothesized that CD133 positive cells, representing the small population of cancer stem cells, would demonstrate resistance to traditional chemotherapeutic agents. In order to test this hypothesis, WST-1 Cell Proliferation Assay was used to examine the drug sensitivity of CD133 positive cells and CD133 negative cells, which were collected by FACS sorting from the three primary cultured cells (No. 66, No. 377 and No. 1049). Both CD133 positive and negative cells were exposed to conventional chemotherapeutic agents, temozolomide, carboplatin, VP16 or Taxol at various concentrations, for up to 48 hours in 10% FBS/DMEM/F-12 medium. We found that CD133 positive cells isolated from the above three cell lines showed dramatic drug resistance to the four agents including temozolomide, carboplatin, VP16 and Taxol at different concentrations compared to autologous CD133 negative cells. One representative experiment on each primary cultured cell line was shown in Fig. 3, Fig. 4 and Fig. 5, respectively.

### Expression of genes related to drug resistance and inhibiting cell apoptosis

In order to address the mechanism of CD133 positive tumor cells showing strong resistance to chemotherapeutic drugs, both CD133 positive cells and CD133 negative were collected by FACS sorting from the three primary cultured cells (No. 66, No. 377 and No. 1049) and used real-time PCR to investigate the expression of multi-drug resistance and DNA mismatch repair related genes, as well as genes related to inhibiting cell apoptosis within these two populations. BCRP1 has been demonstrated to play an important role in the drug resistance of normal stem cells and tumor stem cells [6,7]. In addition, the presence of the DNA repair protein MGMT has been demonstrated to render cells resistant to cytotoxic actions of methylating and chloroethylating agents, such as temozolomide [8,9]. As shown in Table 1 section B, for example, the average level of BCRP1 and MGMT was increased to 6.5 and 32.4 times in CD133 positive cells from No.66 when compared to those of autologous CD133 negative cells, respectively. Furthermore, anti-apoptotic genes, including FLIP, BCL-2 and BCL-XL, were also found at significantly higher levels in CD133 positive cells than in autologous CD133 negative cells (e.g. average 294, 13.9 and 5.6 times increase in CD133 positive cells from No.66, respectively). The inhibitor of apoptosis protein (IAPs) family blocks cell death. IAP family members, XIAP, cIAP1, cIAP2, NAIP and survivin are found at higher expression levels on CD133 positive cells than those in CD133 negative cells (e.g. average 21.9, 39.0, 3.0, 12.1, and 1.6 times increase in CD133 positive cells from No.66, respectively). It has been demonstrated that SIRT1 deacetylates the DNA repair factor Ku70, causing it to sequester the pro-apoptotic factor, BAX, away from mitochondria, thereby inhibiting stress-induced apoptotic cell death [10]. SIRT1 deacetylase mRNA expression is increased average 4.9 times, however, the pro-apoptotic gene BAX is decreased about 3 times in CD133 positive cells from No.66 when compared to autologous CD133 negative cells. Overall, the patterns of gene expression are very similar in CD133 positive cells derived from both cell lines from No. 377 and No. 1049. These results were consistent and supportive with the results of chemoresistance, which CD133 positive cells showed significant resistance to four common chemotherapeutic drugs compared to autologous CD133 negative cells.

### Analysis of CD133 expression on glioblastoma tumor tissue

**Figure 6:**
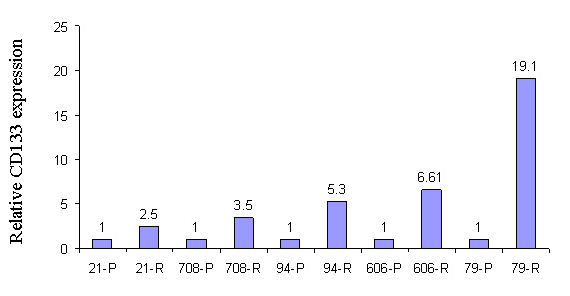
**CD133 mRNA expression in primary (P) and recurrent (R) tumor tissue**. Total RNA was extracted from both primary and recurrent tumor tissue derived from five patients. CD133 mRNA expression was measured by real-time qPCR. The relative CD133 mRNA level of recurrent tumor was presented as the fold increase compared to that of autologous primary tumor tissue (see Methods). Data are representative of two independent experiments.

Malignant glioma is a highly recurrent tumor even after surgery, chemotherapy, radiation and immunotherapy. We hypothesized that there is a small population of cancer stem cells that are resistant to current glioblastoma therapies and play a very important role in tumor recurrence. We further hypothesized that recurrent tumors should contain more cancer stem cells than primary tumor, because recurrent tumors are relatively resistant to currently available therapies. In order to address the potential role of CD133 positive tumor cells in glioblastoma recurrence, we compared the CD133 expression upon first and second resection of tumor tissue from the same patient. Five pathological confirmed grade IV astrocytoma (GBM) patients underwent an initial operation at our institution to obtain the primary tumor tissue, then re-operation following radiation, chemotherapy, and/or immunotherapy to obtain recurrent tumor tissue. As Fig. 6 shows, for all tested patients, CD133 expression was significantly higher in recurrent tumor tissue than that in autologous primary tumor tissue, respectively.

## Discussion

Although the biological function of CD133 is not well understood, the CD133 currently serves as a useful marker for the isolation of brain cancer stem cells [1,3]. The identification of brain cancer stem cells provides a powerful tool for the investigation of the tumorigenic process in the central nervous system, and will be crucial in developing therapies that use brain cancer stem cells as a target [11].

Our data indicated that a large number of CD133 positive cancer stem cells could be maintained without differentiation in 10% FBS culture medium after 3–6 passages. Recently, Kondo and his colleagues found that C6, an established malignant murine glioma cell line (which has been maintained for years in culture) contained a subpopulation of cancer stem cells [12]. Furthermore, a distinct "side population" of cells with high drug efflux capacity were also found in the human glioblastoma cell line U-87, which is consistent with the findings of the study of C6 glioma cells [7]. We propose that a minor subpopulation of cancer stem cells derived from glioblastoma tumor tissue may be maintained even in FBS containing medium which may be a significant character of cancer stem cells from normal stem cells.

Recent studies show that cancer and normal stem cells share the same self-renewal mechanisms, such as the Bmi-1 and Wnt canonical pathways [13,14]. In the present study, we found thatCD133 positive cells expressed higher levels of CD90, CD44, Nestin, Msi1, MELK, GLI1 and PTCH. Particularly, Bmi-1, PSP, SHH, OCT4 and Snail were only expressed on CD133 positive cells; none of the five genes were detectable on CD133 negative tumor cells. The data suggests that these genes involved in self-renewal are expressed in CD133 positive cancer stem cells while not in CD133 negative cells. However, Hemmati *et al*. examined mRNA expression profiles of differentiated and undifferentiated spheres from six different brain tumours and found higher levels of musashi-1, Sox2, MELK, PSP, Bmi-1, and Nestin were not strictly associated with the undifferentiated spheres but differed strongly among the different specimens and these genes were sometimes even stronger expressed in the differentiated [4].

Bmi-1 functions as a transcriptional repressor that targets the INK4a locus and is necessary for self-renewal in the blood and brain. Parallel to these normal organ systems, Bmi-1 can also play an integral role in the malignant transformation of the HOX A9/MEIS – induced murine leukemia model [13] as well as in tumors of neural origin [15]. These findings highlight the notion that the molecular regulation of self-renewal can be observed in both normal and cancer stem cells. Our observation of Bmi-1, SHH, Oct4, PSP and Snail expression only in the cancer stem cell component of a human brain malignancy may shed light on the dynamic nature of the cancer stem cell to exit the capacity to self renew as it differentiates into a daughter cell.

Cancer stem cells may be responsible for the initiation and maintenance of neoplastic tissue. Alternatively, neural progenitor or terminally differentiated cells may dedifferentiate to a stem-like state to form the tumor initiating cell. The tumor initiating and sustaining stem-like cells' resistance to chemo- and radiation therapy, may explain why such traditional therapies can shrink a tumor but often cannot completely eradicate it resulting in eventual recurrence[16]. In this study, we tested four common chemotherapeutic agents on CD133 positive cancer stem cells. Consistent with our hypothesis, CD133 positive cancer stem cells are significantly resistant to four tested chemotherapeutic agents, including temozolomide, carboplatin, VP16 and Taxol than autologous CD133 negative cells.

Temozolomide is a new oral alkylating agent that is widely used for high-grade gliomas [17]. The cytotoxicity of temozolomide is primarily due to the formation of O^6^-methylguanine in DNA, which mispairs with thymine during the next cycle of DNA replication [18]. Subsequent futile cell cycles of DNA lead to mismatched repairs and result in cancer cell death. [19]. Current available data generated from investigations in cell lines and tumor samples, suggest that resistance to temozolomide is significantly linked to MGMT-mediated DNA repair in high-grade gliomas, primitive neuroectodermal tumors, and ependymomas [20]. Our study showed CD133 positive cells had significant resistance to temozolomide compared to autologous CD133 negative cells, which was consistent with the results of higher MGMT expression in CD133 positive cells.

One of the important mechanisms of drug resistance is the expression of ATP-binding cassette transporter protein, such as BCRP1. BCRP1 accounts for chemoresistance of some clinical cancers including acute myeloid leukemia, non-small cell cancer, and breast cancer. In both breast carcinoma and acute myeloid leukemia, putative cancer stem cells have been isolated and characterized. The evidence suggests that the "side population (SP)" phenotype is associated with high expression levels of BCRP1 [6]. The exclusion of Hoechst 33342 dye defines the pluripotential side population (SP) originally reported in hematopoietic stem cells. Their high drug efflux capacity correlates with the strong expression of the drug-transporter protein BCRP1. Therefore, SP has become a common approachto identify putative adult stem cells [21] and cancer stem cells [12]. BCRP1 has been shown to be specifically expressed in the fetal neural stem cell by microarray and immunocytochemical analysis [22]. We found that CD133 positive cells express higher levels of BCRP1, which indicate that BCRP1 may also play an important part in the drug resistance of CD133 positive cells. BCRP1 over-expressing tumor cells, however, are only resistant to mitoxantrone, adriamycin, daunorubicin, etoposide, topotecan, and irinotecan. They are not resistant to Taxol and vincristine [23]. In the present study, CD133 positive cells derived from three cell lines are all resistance to carboplatin and Taxol. Therefore, we proposed that both anti-apoptosis factors and BCRP1 contribute the drug resistant property on CD133 positive cancer stem cells.

Apoptosis mechanisms are hypothesized to play an important role in the resistance of tumor cells to chemotherapy [24]. Most studies indicate that increased levels of the anti-apoptotic protein Bcl-2 correlates with chemotherapy resistant disease and decreased overall survival [25]. The inhibitor of apoptosis protein (IAPs) family are anti-apoptotic proteins that bind and inhibit caspases 3, 7 and 9, and thereby prevent apoptosis [26]. Growing evidence also indicates that IAPs also modulate cell division, cell cycle progression and signal transduction pathways [26]. In this study, we found that Bcl-2, FLIP, BCL-XL and all of the IAPs we tested, including Class I IAPs (XIAP, cIAP1 and cIAP2); Class II IAP (NAIP) and Class III IAP (survivin) expressed higher mRNA levels in CD133 positive cells than in CD133 negative cells. The data supports the hypothesis that apoptosis pathways contribute to drug resistance of CD133 positive cancer stem cells.

Bcl-2 promotes prolonged but not indefinite cell survival under apoptotic stress from radiation, chemotherapy drugs, and toxins [27]. The Bcl-2 family is made up of both suppressors (e.g. Bcl-2, Bcl-_XL _and MCL-1) and inducers (e.g. Bax, Bad, Bak and Bid) of apoptosis. AML leukemic cells recovered from patients with minimal residual disease have demonstrated high levels of Bcl-2 and Bcl-_XL _[28], suggesting a significant role of these genes in resistance to therapy.

ATRA has been shown to increase sensitivity to ARA-C by downregulating Bcl-2 and Bcl-_XL _in vitro [29]. As a clinical corollary, retinoids given with chemotherapy can improve the survival of AML patients. Bcl-2 expression in patient samples of AML cells correlated with decreased rates of remission and event-free survival [30]. Furthermore, a low Bcl-2/Bax ratio was associated with prolonged survival [31]. These findings support the notion the inhibition of apoptosis is directly associated with response to chemotherapy and hence clinical outcome.

Neural stem cells (NSCs) express the markers nestin and CD133, and differentiate into neurons, astrocytes and oligodendrocytes at a clonal level [32]. An important characteristic of NSCs, not fully understood, is their migratory ability and their tropism to brain pathology. High expression levels of functional chemokine receptor CXCR4 have been found on human neural stem cells [33]. These findings suggest that CXCR4 may play a significant role in directing NSC migration during CNS development. Similar to normal NSCs, CD133 positive cancer stem cells showed 337.8 times increase on the expression levels of CXCR4 than did CD133 negative cells, which suggests CD133 positive cancer stem cells have higher capability of migration and may play an important role in glioma invasion.

Finally, CD133 expression levels are higher in recurrent tumor tissue than in autologous primary tumor tissue, which suggests that CD133 positive cancer stem cells may play an important role in the tumor's ability to resist chemotherapies and radiation therapy.

Taken together, because of higher BCRP1, MGMT and IAPs levels, CD133 positive cancer stem cells may be resistant to conventional chemotherapy and contribute to disease relapse. Since normal tissue stem cells and cancer stem cells have many similar properties, further studies should focus on the difference between CD133positive normal tissue stem cells *versus *CD133 positive cancer stem cells in order to find unique pathways to differentiate these two cell populations. These differences may be exploited with the aim of targeting the CD133 positive cancer stem cell population either biochemically or immunologically without harming normal tissue stem cells. Delineating the molecular genetics and epigenetics involved in the propagation and differentiation of these stem cell populations will be useful in defining the therapeutic window to target only the cancer stem cell.

## Materials and methods

### Tumor specimens and primary cell culture

Glioblastoma specimens were obtained from patients (with informed consents) via the Brain Tumor Registry and were reviewed and released by a pathologist in the operating room. Independent pathologists classified the tumors by type and grade in accordance with the WHO histological grading of central nervous system tumors. IRB certified technicians processed the glioma tissues under sterile conditions in a laminar flow hood. Tumor cells were cultured in the following complete medium: Ham's DMEM/F-12 with high glucose (Irvine scientific, Santa Ana, CA), 10 mM HEPES (Invitrogen, Carlsbad, CA), 0.1 mg/ml Gentamicin (Invitrogen) and 10% heat-inactivated FBS (Irvine Scientific, Santa Ana, CA). The cultured cells were maintained for 3–6 passages. Three adult glioblastoma primary cultured cell lines (No. 66, No. 377 and No.1049) were used to isolate CD133 positive cancer stem cells by FACS sorting for further experiments. In order to investigate the capacity of self-newal and clonogenic potential of CD133+ cell, a single isolated CD133 positive cancer stem cell was cultured in a defined serum-free NSC medium[34] containing 20 ng/ml of basic fibroblast growth factor (bFGF, Peprotech, Rocky Hill, NJ) and 20 ng/ml of epidermal growth factor (EGF, Peprotech). Both primary and recurrent glioblastoma tissue from five different patients were used to analyze CD133 expression on tumor tissue.

### Chemotherapeutic agents

Temozolomide was kindly supplied by the Schering-Plough Research Institute (Kenilworth, NJ) and was dissolved in DMSO (Sigma Chemical Co., St Louis, MO) at 100 mM stock solution. Carboplatin, etoposide (VP16) and paclitaxel (Taxol) were obtained from Sigma-Aldrich (St. Louis, MO).

### Immunofluoresence staining

To examine CD133 protein expression, a marker of cancer stem cells, immunofluoresence staining was performed as previously described [34]. Briefly, the cells growing in pre-coated chamber slides were fixed with 2% paraformaldehyde for 15 min at room temperature, treated with 10% normal goat serum, and then stained with anti-CD133 antibody (rabbit polyclonal IgG; 1: 100; Abcam Inc, Cambridge, MA). The primary antibodies were detected with Alexa fluor 568 goat anti-rabbit IgG, (1: 1000; Molecular Probes, Eugene, Oregon).

### Flow cytometry analysis and FACS sorting CD133 positive cells

Tumor cells were collected and stained with anti-CD133 antibody (mouse monoclonal IgG1; 1: 10; Milteny Biotec) or IgG1 isotype control antibody (BD Pharmingen, San Diego, CA). After PE-ant-mouse IgG1 (BD Pharmingen) staining for 30 min, CD133 staining was analyzed by flow cytometry FACSCalibur (Becton Dickinson, San Jose, CA). CD133 positive cells were sorted by DAKO cytomation (DAKO, Carpinteria, CA).

### RNA isolation and cDNA synthesis

Total RNA was extracted from fresh tumor tissue and isolated CD133 positive and CD133 negative cells using an RNA4PCR kit (Amibion, Austin, TX) according to the manufacturer's protocol. For cDNA synthesis, ~1 μg total RNA was reverse-transcribed into cDNA using Oligo dT primer and iScript cDNA synthesis kit reverse transcriptase. cDNA was stored at -20°C for PCR.

### Real-time Quantitative RT-PCR

Gene expression was quantified by real-time quantitative RT-PCR using QuantiTect SYRB Green dye (Qiagen, Valencia, CA). DNA amplification was carried out using Icycler (BIO-RAD, Hercules, CA), and the detection was performed by measuring the binding of the fluorescence dye SYBR Green I to double-stranded DNA. All the primer sets were provided by Qiagen (see Additional file 1). The relative quantities of target gene mRNA against an internal control, beta-actin, was possible by following a ΔC_T _method. An amplification plot that had been the plot of fluorescence signal *vs*. cycle number was drawn. The difference (ΔC_T_) between the mean values in the duplicated samples of target gene and those of beta-actin were calculated by Microsoft Excel and the relative quantified value (RQV) was expressed as 2−ΔCT
 MathType@MTEF@5@5@+=feaafiart1ev1aaatCvAUfKttLearuWrP9MDH5MBPbIqV92AaeXatLxBI9gBaebbnrfifHhDYfgasaacH8akY=wiFfYdH8Gipec8Eeeu0xXdbba9frFj0=OqFfea0dXdd9vqai=hGuQ8kuc9pgc9s8qqaq=dirpe0xb9q8qiLsFr0=vr0=vr0dc8meaabaqaciaacaGaaeqabaqabeGadaaakeaacqaIYaGmdaahaaWcbeqaaiabgkHiTiabfs5aejabboeadnaaBaaameaacqqGubavaeqaaaaaaaa@3287@. The relative expression of each gene or sample presented in this report was compared to autologous CD133 negative cells or recurrent tumor tissue.

### Drug cytotoxicity assay

The number of viable cells following drug treatment was assessed using a WST-1 Cell Proliferation Assay (Roche, Indianapolis, IN). 1 × 10^4 ^cells/well were plated in 96-well plates, then allowed to attach overnight and finally chemotherapeutic agents at various concentrations were added for 48 h in 10% FBS/DMEM/F-12 culture medium. Four hours prior to harvest, 20 μl/well of the reagent WST-1 was added and incubated for 1.5 h at 37°C. An increase in the number of viable cells resulted in an increase in the overall activity of mitochondrial dehydrogenases in the sample with an ensuing increase in formazan dye formation. The quantity of formazan dye was quantified by measuring the optical density of the dye solution at 450 nm with a scanning multiwell spectrophotometer (Molecular Devices, Sunnyvale, CA) using 890 nm as the internal reference [35]. All results in the study were based on at least eight parallel measurements each time and each measurement was repeated in up to two independent experiments.

### Statistical analysis

All the WST-1 assay results were analyzed by one way analysis of variance (ANOVA) followed by post-hoc (Tukey) tests using Sigmastat for Windows, version 2.03. A level of P < 0.05 was considered statistically significant.

## Supplementary Material

Additional file 1 The sequences of primers used for SYBR Green real-time PCR. Twenty-six sets of primers were carefully designed to test twenty-six different genes mRNA expression in CD133+ and CD133- cells. Click here for file
