# Supplementary material for: Analysis of gene expression and chemoresistance of CD133+ cancer stem cells in glioblastoma
Source: Mol Cancer. 2006 Dec 2;5:67. doi: 10.1186/1476-4598-5-67 (PMC1697823; doi:10.1186/1476-4598-5-67)
Supplement: Additional file 1 — The sequences of primers used for SYBR Green real-time PCR. Twenty-six sets of primers were carefully designed to test twenty-six different genes mRNA expression in CD133+ and CD133- cells. [file 1476-4598-5-67-S1.doc]

The sequences of primers used for SYBR Green real-time PCR

| Gene | Forward | Reverse |
| --- | --- | --- |
| Beta-actin | 5’-TTCTACAATGAGCTGCGTGTG-3’ | 5’-GGGGTGTTGAAGGTCTCAAA-3’ |
| CD133 | 5’-GCATTGGCATCTTCTATGGTT-3’ | 5’-CGCCTTGTCCTTGGTAGTGT-3’ |
| MSI1 | 5'-GAGACTGACGCGCCCCAGCC-3' | 5'-CGCCTGGTCCATGAAAGTGACG-3' |
| MELK | 5'-CTTGGATCAGAGGCAGATGTTTGGAG-3' | 5'-GTTGTAATCTTGCATGATCCAGG-3' |
| PSP | 5'-GGCGGGGCAGTGCCTTTCAAA-3' | 5'-TGTTGGCTGCGTCTCATCAAAACC-3' |
| CD90 | 5’-CGCTCTCCTGCTAACAGTCTT-3’ | 5’-CAGGCTGAACTCGTACTGGA-3’ |
| Nestin | 5-ATCGCTCAGGTCCTGGAA-3’ | 5’-AAGCTGAGGGAAGTCTTGGA-3’ |
| CD44 | 5’-AGAAGGTGTGGGCAGAAGAA-3’ | 5’-AAATGCACCATTTCCTGAGA-3’ |
| GLI1 | 5’-AGGGAGGAAAGCAGACTGAC-3’ | 5’-CCAGTCATTTCCACACCACT-3’ |
| CXCR4 | 5’-GATCAGCATCGACTCCTTCA-3’ | 5’-GGCTCCAAGGAAAGCATAGA |
| Bmi-1 | 5'-GGAGACCAGCAAGTATTGTCCTTTTG-3' | 5'-CATTGCTGCTGGGCATCGTAAG-3' |
| PTCH | 5’-CGCCTATGCCTGTCTAACCATGC-3’ | 5’-AAATGGCAAAACCTGAGTTG-3’ |
| OCT4 | 5’-CCTGAAGCAGAAGAGGATCA-3’ | 5’-CCGCAGCTTACACATGTTCT-3’ |
| SHH | 5’-GGACAGGCTGATGACTCAGA-3’ | 5’-GCCCTCGTAGTGCAGAGACT-3’ |
| Snail | 5'-ACCACTATGCCGCGCTCTT-3' | 5'-GGTCGTAGGGCTGCTGGAA-3' |
| SIRT1 | 5'-ACTTGTACGACGAAGACGAC-3' | 5'-CAGAAGGTTATCTCGGTACC-3' |
| Survivin | 5′-TGCCTGGCAGCCCTTTC-3′ | 5′-CCTCCAAGAAGGGCCAGTTC-3 |
| cIAP1 | 5′-CAGCCTGAGCAGCTTGCAA-3′ | 5′-CAAGCCACCATCACAACAAAA-3′ |
| cIAP2 | 5′-TCCGTCAAGTTCAAGCCAGTT-3′ | 5′-TCTCCTGGGCTGTCTGATGTG-3’ |
| NAIP | 5′-GCTTCACAGCGCATCGAA-3′ | 5′-GCTGGGCGGATGCTTTC-3′ |
| XIAP | 5′-AGTGGTAGTCCTGTTTCAGCATCA-3′ | 5′-CCGCACGGTATCTCCTTCA-3′ |
| BCL-2 | 5′-CATGCTGGGGCCGTACAG-3 | 5′-GAACCGGCACCTGCACAC-3′ |
| BCL-X_L_ | 5′-TGCATTGTTCCCATAGAGTTCCA-3′ | 5′-CCTGAATGACCACCTAGAGCCTT-3′ |
| FLIP | 5′-CATCCACAGAATAGACCTGAAGACAA-3′, | 5′-GCTTGGAGAACATTCCTGTAACTTG-3′, |
| BAX | 5'-TGG AGCTGCAGAGGATGATTG-3' | 5'-GAAGTTGCCGTCAGAAAACATG-3' |
| BCRP-1 | 5'-TGGCTGTCATGGCTTCAGTA-3' | 5'-GCCACGTGATTCTTCCACAA-3' |
| MGMT | 5’-CTGGCTGAATGCCTACTTCC-3’ | 5’- CAACCTTCAGCAGCTTCCAT-3’ |
